# Chinese Herbal Medicine Qi Ju Di Huang Wan for the Treatment of Essential Hypertension: A Systematic Review of Randomized Controlled Trials

**DOI:** 10.1155/2013/262685

**Published:** 2013-06-25

**Authors:** Jie Wang, Xingjiang Xiong, Guoyan Yang, Yuqing Zhang, Yongmei Liu, Yun Zhang, Zhenpeng Zhang, Jun Li, Xiaochen Yang

**Affiliations:** ^1^Department of Cardiology, Guang'anmen Hospital, China Academy of Chinese Medical Sciences, Beijing 100053, China; ^2^Centre for Evidence-Based Chinese Medicine, Beijing University of Chinese Medicine, Beijing 100029, China; ^3^Department of Clinical Epidemiology and Biostatistics, McMaster University, ON, Canada L8S 4L8

## Abstract

*Background*. Chinese herbs are potentially effective for hypertension. Qi Ju Di Huang Wan (QJDHW) is a commonly used Chinese herbal medicine as a monotherapy or in combination with other antihypertensive agents for the treatment of essential hypertension (EH). However, there is no critically appraised evidence such as systematic reviews or meta-analyses on the effectiveness and safety of QJDHW for EH. *Methods and Findings*. CENTRAL, PubMed, CBM, CNKI, VIP, and online clinical trial registry websites were searched for published and unpublished randomized controlled trials (RCTs) of QJDHW for essential hypertension up to January 2013 with no language restrictions. A total of 10 randomized trials involving 1024 patients were included. Meta-analysis showed that QJDHW combined with antihypertensive drugs was more effective in lowering blood pressure and improving TCM syndrome for the treatment of essential hypertension than antihypertensive drugs used alone. No trials reported severe adverse events related to QJDHW. *Conclusions*. Our review suggests that QJDHW combined with antihypertensive drugs might be an effective treatment for lowering blood pressure and improving symptoms in patients with essential hypertension. However, the finding should be interpreted with caution because of the poor methodological quality of included trials. There is an urgent need for well-designed, long-term studies to assess the effectiveness of QJDHW in the treatment of essential hypertension.

## 1. Introduction

Hypertension is an increasingly prevalent chronic condition that is associated with serious morbidity and mortality. It is an important risk factor for the development and progression of cardiovascular disease, which is predicted that it will become the leading cause of death and disability worldwide by 2020 [1]. Hypertension is classified as either essential hypertension (EH) or secondary hypertension, and EH accounts for about 90–95% of the cases characterized by high blood pressure with no obvious underlying medical causes [2]. In developing countries, it is a major medical concern that the high rate of undetected and untreated EH [3]. In China, the prevalence of EH is currently 18.8% [4]. The high rates of EH, especially undiagnosed EH, throughout the oriental countries double the risk of cardiovascular diseases, including coronary heart disease, congestive heart failure, ischemic and hemorrhagic stroke, renal failure, and peripheral arterial disease [5]. 

There is a growing tendency for people to turn to complementary and alternative medicine (CAM) [6, 7]. In addition, the increasing prevalence of hypertension creates a broad market for alternative therapy to aid the management of blood pressure [8]. Several CAM clinical studies, including a substantial number of randomized controlled trials (RCTs) and systematic reviews, have shown that CAM is effective and safe for the treatment of hypertension [9–12]. It can also improve appetite, intestinal motility, metabolism, and emotional factors such as stress. Furthermore, studies showed that the application of CAM could enable tailored therapy in clinical practice, including lifestyle modification and individual choice of drugs lowering blood pressure [13, 14].

Qi Ju Di Huang Wan (QJDHW) decoction containing eight commonly used herbs (Chinese wolfberry fruit, chrysanthemum flower, common yam rhizome, tree peony bark, water plantain rhizome, *cornus*, and poria) had been used to relief symptoms like dizziness and vertigo for thousands of years in China. From the perspective of traditional Chinese medicine, it is believed that the mechanism of QJDHW may be related to calming the liver, suppressing liver yang hyperactivity, and nourishing kidney yin. Biochemically, QJDHW showed a good effect in decreasing the concentrations of angiotensin in plasma and myocardium, reducing the endothelin (ET) content and improving kidney blood stream in rats with essential hypertension [15–17].

Currently, it is common to see patients with essential hypertension seek QJDHW used alone or combined with antihypertensive agents as an alternative method. Recent studies also showed that QJDHW could help to control blood pressure [18, 19]. However, the evidence examining the effectiveness of QJDHW for essential hypertension has never been systematically summarized. Thus, we performed this systematic review to critically assess the effectiveness of QJDHW for the treatment of essential hypertension. 

## 2. Methods

### 2.1. Search Strategy

We searched the following sources up to January 2013: the Cochrane library, including the Cochrane Central Register of Controlled Trials (CENTRAL, 2012), PubMed, Chinese bases, including Chinese Biomedical Literature Database (CBM), Chinese National Knowledge Infrastructure (CNKI), and Chinese Scientific Journal Database (VIP). In addition, we also searched the databases of clinical trials such as Current Controlled Trials (http://www.controlled-trials.com/isrctn/), the National Centre for Complementary and Alternative Medicine (NCCAM) at the National Institutes of Health (NIH) (http://www.nccam.nih.gov/), and the Complementary and Alternative Medicine Specialist Library at the NHS National Library for Health (http://www.library.nhs.uk/cam/). The searching terms were “Qi Ju Di Huang Wan”, “Yuan Fa Xing Gao Xue Ya (essential hypertension)”, and “Gao Xue Ya (hypertension)”. No language restriction was applied.

### 2.2. Inclusion Criteria and Exclusion Criteria

Our paper was restricted to RCTs that compared QJDHW or modified QJDHW, regardless of the preparation, with conventional antihypertensive drugs. RCTs comparing QJDHW combined antihypertensive drugs with antihypertensive drugs were also included. Quasi-RCTs were not considered. Animal studies, clinical trials including case report, case series traditional reviews were excluded. The main outcome measure was blood pressure (BP). The other outcome measures included the *Scale for TCM Syndrome and Symptom Differentiation* (TCM-SSD), the level of blood lipids (BL), plasma viscosity (PV), angiotensin II (AngII), endothelin (ET), calcitonin gene-related peptide (CGRP), and safety. For TCM-SSD, the effect was presented as markedly effective, effective, and ineffective. Markedly effective was defined as the main symptoms such as headache, dizziness, palpitations, insomnia, tinnitus, and irritability disappeared or the TCM-SSD scores reduced rate ≥70%; effective was defined as the main symptoms relieved or 70% > TCM-SSD scores reduced rate ≥30%; ineffective was defined as the main symptoms that do not change or the TCM-SSD scores reduced rate <30%.

### 2.3. Study Selection and Data Extraction

The titles and abstracts of potentially relevant references were identified through the literature search and reviewed independently by 2 reviewers (G. Yang and Y. Zhang) according to predefined criteria. Discrepancies were resolved by consensus with another investigator (J. Wang). The following data were extracted: (1) citations (authors of study, year of publication), (2) methodological information, (3) participants information (sample size, age), (4) detailed information of interventions and controls, (5) outcome measures, and (6) adverse events. 

### 2.4. Trial Quality Assessment

We assessed the methodological quality of included RCTs using Cochrane risk of bias tool. It has the following six domains: random sequence generation (selection bias), allocation concealment (selection bias), blinding of participants and personnel (performance bias), blinding of outcome data (attrition bias), incomplete outcome data (attrition bias), and selective reporting (reporting bias). The judgment was given as “high risk”, “unclear risk”, or “low risk”: trials that met all the criteria were categorized as low risk of bias; those that met none of the criteria were categorized as high risk of bias; the others were categorized as an unclear risk of bias if insufficient information was available to make a judgment. Disagreements were resolved by discussion.

### 2.5. Data Analysis

The statistical package (RevMan 5.1.7) provided by Cochrane Collaboration was used for data analyses. Dichotomous data were expressed as risk ratio (RR) and continuous outcomes as weighted mean difference (WMD), with their 95% confidence intervals (CI), respectively. Meta-analysis was performed if the intervention, control, and outcome were the same or similar. The statistical heterogeneity was examined with the *I* ^2^-test, where *I* ^2^ values of 50% or more were considered to be an indicator of a substantial heterogeneity. In the absence of significant heterogeneity, we pooled data using a fixed-effect model (*I* ^2^ < 50%); otherwise we used random effects model (*I* ^2^ > 50%) [20]. To maximize the similarities among studies that would be combined, and data were further stratified where possible into subgroups based on different types of interventions.

## 3. Result

### 3.1. Description of Included Trials

**Figure 1:**
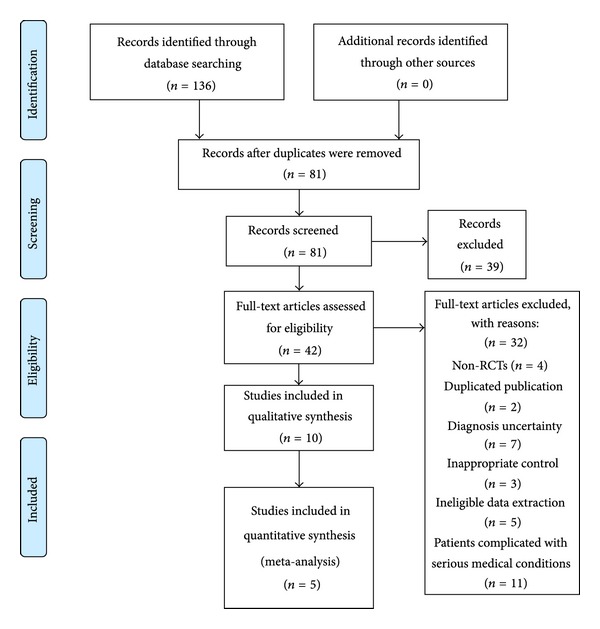
Flow Diagram of the literature searching and study selection.

We identified 136 potentially relevant references from the electronic and manual searches. After screening the titles and abstracts, 94 studies were excluded because of duplicated publication (55 studies), animal studies (4 studies), and noncontrolled clinical trials (35 studies) including case report, case series, and traditional review. Full texts of 42 papers were retrieved, and finally 10 RCTs [21–30] were included. A total of 5 RCTs [21–25] were included in meta-analysis. All the RCTs were conducted in China and published in Chinese. The search for ongoing registered trials identified no trials (Figure 1).

The characteristics of the 10 RCTs are summarized in Table 1. The total number of participants with essential hypertensive was 1024. The age of participants varied from 32 to 80 years. Five different diagnostic criteria of hypertension were used in the included trials: one trial [21] used 1999 WHO-ISH guidelines for the management of hypertension (1999 WHO-ISH GMH), one trial [23] used Seventh Report of the Joint National Committee on the Prevention, Detection, Evaluation, and Treatment of High Blood Pressure (JNC 7), one trial [22] used China Guidelines on Prevention and Management of High Blood Pressure-2005 (CGPMHBP-2005), one trial [28] used CGPMHBP-2000, one trial [26] used 1978 WHO-ISH guidelines for the management of hypertension (1999 WHO-ISH GMH), and five trials [24, 25, 27, 29, 30] only demonstrated patients with essential hypertension. Of the 10 trials, three trials [21, 23, 26] reported TCM diagnostic criteria with yindeficiency and excessive yang syndrome and used Guidelines of Clinical Research of New Drugs of Traditional Chinese Medicine (GCRNDTCM); one trial [25] only demonstrated patients with yindeficiency and excessive yang syndrome in TCM, and six trials [22–24, 27–30] have not reported TCM diagnostic criteria.

The interventions included Qi Ju Di Huang Wan in preparation of (modified) decoction and pill. The compositions of different QJDHW preparations are presented in Table 2. Of the 10 trials, 4 trials [21, 26, 27, 30] investigated the preparation of “Qi Ju Di Huang Wan” used alone versus antihypertensive drugs, and the rest 6 trials [22–25, 28, 29] compared the preparation of “Qi Ju Di Huang Wan” plus antihypertensive drugs versus antihypertensive drugs. The treatment duration ranged from 4 to 24 weeks. 

### 3.2. Methodological Quality of Included Trials

**Figure 2:**
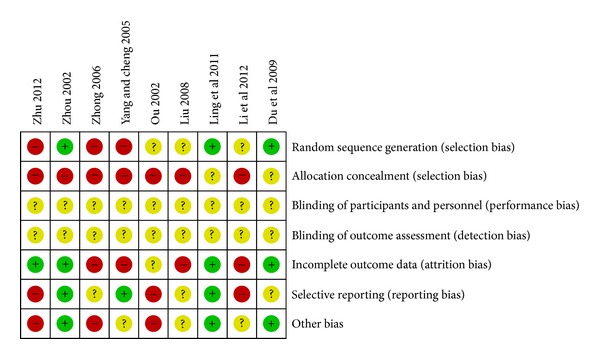
Risk of bias summary: review authors' judgments about each risk of bias item for each included study.

**Figure 3:**
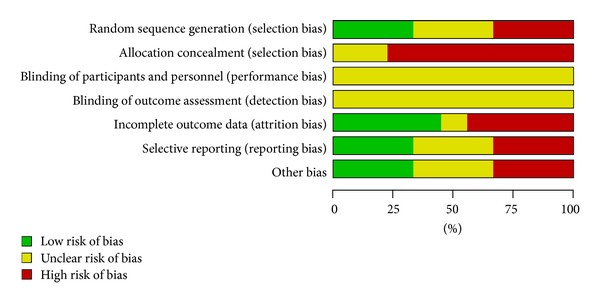
Risk of bias graph: review authors' judgments about each risk of bias item presented as percentages across all included studies.

The methodological quality of the 10 trials was generally low. All of the 10 included trials mentioned the randomized allocation of participants, and only two trials [23, 27] stated the methods of sequence generation of random number table. However, insufficient information was provided to judge whether it was conducted properly or not. Among the 10 trials, allocation concealment and double blind were not mentioned. None of the trials reported a dropout or withdrawae, and none of the trials reported sample size calculation. Since the protocols of all the 10 included trials were not accessible, selective reporting was generally unclear. In addition, no trial reported a followup (Figures 2 and 3). 

### 3.3. Effect of the Interventions

#### 3.3.1. “Qi Ju Di Huang Wan” versus Antihypertensive Drugs (Western Medicine)

 Four trials [21, 26, 27, 30] compared the preparation of “Qi Ju Di Huang Wan” used alone with antihypertensive drugs. 

*Blood Pressure*. There was no significant difference between the two groups in systolic blood pressure (WMD: 0.10 [−3.38, 3.58]; *P* = 0.96) and diastolic blood pressure (WMD: −0.20 [−2.42, 2.02]; *P* = 0.86) after 8 weeks of treatment (see Tables 3 and 4). 

*TCM-SSD Scores. *One trial [21] reported the TCM-SSD scores. The result showed a significant difference between QJDHW and antihypertensive drugs (*P* = 0.02). 

#### 3.3.2. “Qi Ju Di Huang Wan” Plus Antihypertensive Drugs versus Antihypertensive Drugs

 Six trials [22–25, 28, 29] compared the combination of modified QJDHW plus antihypertensive drugs with antihypertensive drugs. 

**Figure 4:**
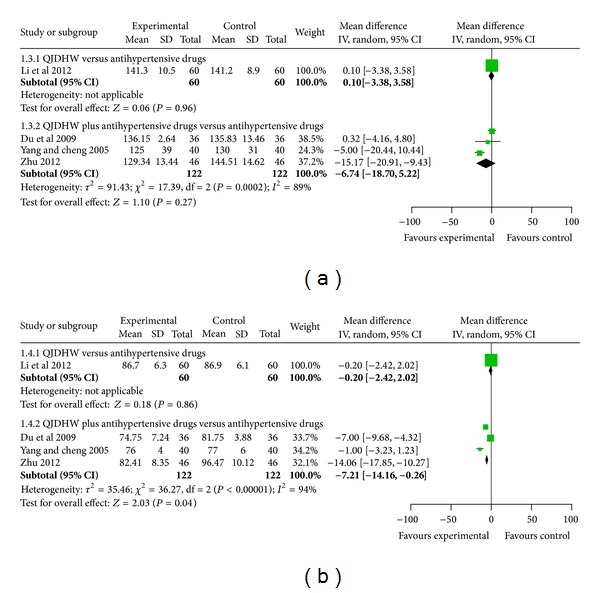
The forest plot of comparison of two groups for the outcome of blood pressure: (a) outcome of systolic blood pressure, (b) outcome of diastolic blood pressure.

*Blood Pressure*. Three trials [22–24] showed that there was a significant difference between treatment and control groups in systolic blood pressure (WMD: −5.52 [−8.96, −2.08]; *P* = 0.002) and diastolic blood pressure (WMD: −5.26 [−6.83, −3.70]; *P* < 0.00001). The forest plot was shown in the Figure 4. 

*TCM-SSD Scores. *Two trials [23, 25] reported the TCM-SSD scores. The meta-analysis showed that the combination group had a beneficial effect on the improvement of TCM syndrome, compared to the antihypertensive drugs used alone (RR: 1.48 [1.20, 1.82]; *P *= 0. 0.0003). We could not obtain more details of the TCM-SSD scores. So we just conducted an analysis of dichotomous data between groups (see Table 5).

#### 3.3.3. Other Outcomes (BL, PV, AngII, ET, and CGRP)

 One trial [21] showed that after 8 weeks of treatment, the level of blood lipid (BL) and plasma viscosity (PV) decreased significantly (*P* < 0.05) in QJDHW group compared to captopril group. One trial [23] showed that after 4 weeks of treatment, the level of angiotensin II (Ang II), endothelin (ET) decreased significantly (*P* < 0.01) whereas the level of calcitonin gene-related peptide (CGRP) increased significantly (*P* < 0.05) in QJDH pill plus felodipine (plendil) group compared to felodipine (plendil) group. Furthermore, one trial [25] showed that after 8 weeks of treatment, the level of blood lipid (BL) decreased significantly (*P* < 0.05) in QJDH pill plus verapamil group compared to verapamil group.

#### 3.3.4. Sensitivity Analysis, Subgroup Analysis, and Publication Bias

Due to no sufficient number of trials, we failed to conduct sensitivity analysis and subgroup analysis and also failed to perform funnel plot to detect publication bias.

### 3.4. Adverse Effect

Only one trial mentioned the adverse effect in captopril group such as dry cough [21]. Six trials [21–26] reported no side effect in the QJDHW group compared to antihypertensive drugs.

## 4. Discussion

With the acceptance and popularity of CAM, the potential role of herbal remedies in global health care is being increasingly recognized in the recent years. Currently, more and more systematic reviews (SRs) and meta-analysis have been conducted to assess the efficiency of CAM for EH [31–39]. It has made great contributions to the health and well-being of the people for the unique advantages of CAM in preventing and curing diseases, rehabilitation, and health care. To the best of our knowledge, this is the first systematic review and meta-analysis of RCTs for QJDHW in treating essential hypertension. Our review suggests that QJDHW may be effective for the treatment of hypertension. Based on the findings of meta-analyses of blood pressure and TCM-SSD scores, the preparation of “Qi Ju Di Huang Wan” including pill and decoction used alone or combined with antihypertensive drugs may have some beneficial effects on patients with essential hypertension. This review has the following limitations. Firstly, 5 databases have been searched up to January 2013 including the Cochrane library, PubMed, CBM, CNKI, and VIP. In addition, the databases of clinical trials such as Current Controlled Trials, the National Centre for Complementary and Alternative Medicine have also been searched without language restriction. However, all included trials were published in Chinese. 

Secondly, the methodological quality of most included trials is generally low. Details of randomization were unclear. Concealment of allocation and blinding methods were not described, and reports of dropouts and withdrawals were incomplete. There were two trials (RCTs) [23, 27], stated randomization method through random number table. For the other five trials including Zhu 2012, Du et al. 2009, Yang and Cheng 2005, OuYang 2002, and Zhang 1999 [22–26], they only mentioned that “patients were randomized into two groups”, with no detailed information of randomization generation. All of the included trials did not describe the blinding, we could not judge whether there were performance bias and detection bias because of the awareness of the therapeutic interventions for the subjective outcome measures [40–47]. All the included trials used blood pressure as a primary outcome measure, but half of the included trials evaluated the effectiveness with numerical values. The rest of the trials presented the effect as markedly effective, effective, and ineffective. We have tried to contact authors to get further information either by telephone or email. Unfortunately, no replies and information was got. We recommend that future researchers should follow the basic guidelines for reporting clinical trials such as the Consolidated Standards of Reporting Trials (CONSORT) statement.

Thirdly, the treatment duration of most included studies was short, varying from 4 to 8 weeks. Only one trial has a long duration of 24 weeks. Since hypertension is a chronic condition, it is a great concern of patients about the effect of long-term treatment. Indeed, none of the included trials reported the mortality rate or the incidence of complications. Moreover, hypertension may exacerbate with or without treatment, especially in China; a major risk factor for hypertension is unbalanced intake of dietary sodium and potassium. Studies have indicated that high dietary sodium intake may change the circadian rhythm of 24-hour blood pressure, which is characterized by a higher nighttime blood pressure. The prevalence of isolated nighttime hypertension, which is defined as a nighttime systolic/diastolic blood pressure more than 120/70 mm Hg and a daytime systolic/diastolic blood pressure less than 135/85 mm Hg, is higher in Chinese than in Europeans [48, 49]. Therefore, a longer follow-up period with serial measurements of outcomes is suggested to determine the long-term effectiveness of QIDHW prescriptions. Thus, RCTs of QJDHW prescriptions with design to measure the followup of outcomes are urgently needed.

Fourthly, among the included trials, there is inadequate reporting on adverse events. None of the five trials reported information on the adverse effect of QJDHW decoctions or pills. Due to the limited information of adverse events, we could not draw definite conclusions on the safety of QJDHW prescriptions. Though most of Chinese herbal medicines and Chinese patent medicines are widely accepted and safely used, the increasing reports of liver and kidney toxicity and other adverse events related to Chinese medicines [50–59] draw much attention to the concern of safety. We recommend that future clinical trials should use QJDHW prescriptions with caution and report adverse events appropriately.

Fifthly, there may be publication bias in this review. We doubted whether all the RCTs have positive effect of QJDHW prescriptions when analyzed by standard statistical techniques using risk ratios or mean differences. For this, extensive searches for unpublished material have been conducted, but no unpublished “negative” studies were found. 

In general, comparing to three categories (calcium antagonist, beta blocker, and angiotensin-converting enzyme inhibitors) of antihypertensive drugs such as captopril, nifedipine, felodipine (plendil), metoprolol, and verapamil, the preparation of QJDHW appears to lower blood pressure and improve the symptoms with less adverse event. The combination of QJDHW and antihypertensive drugs may have significant effectiveness compared to antihypertensive drugs used alone. However, the quality of RCTs included in our review was limited for us to draw definite conclusions about QJDHW. More rigorous RCTs are required to be performed to prove the effectiveness of QJDHW for treating hypertension.

## 5. Conclusions

Our review indicates that QJDHW for treating essential hypertension has some beneficial effects compared to antihypertensive drugs, although the results are of limited value due to the clinical heterogeneity and low methodological quality of the included studies which prevent us from drawing a definitive conclusion for the effectiveness of QJDHW. However, QJDHW preparations are relatively safe, as a traditional Chinese medical therapy for improving symptoms of essential hypertension. Nevertheless, questions that cannot be conclusively answered at present include whether QIDHW prescriptions should be widely recommended and what the most effective preparation of QJDHW is. More well-designed, long-term clinical trials are needed.

## Figures and Tables

**Table 1:** Characteristics and methodological quality of included studies.

Study ID	Sample	Diagnosis standard	Intervention	Control	Course (week)	Outcome measure
Li et al., 2012 [21]	120	1999 WHO-ISH GMH; GCRNDTCM	QJDHW	Captopril	8	BP; TCM-SSD; BL; PV; side effect
Zhu, 2012 [22]	92	CGPMHBP-2005	QJDH pill plus nifedipine controlled release tablet	Nifedipine controlled release tablet	8	BP
Du et al., 2009 [23]	120	JNC-7; GCRNDTCM	QJDH pill plus felodipine (plendil)	Felodipine (plendil)	4	BP; TCM-SSD; Ang II,ET, CGRP
Yang and Cheng, 2005 [24]	120	Hypertension diagnostic criteria (unclear)	modified QJDHW plus metoprolol	Metoprolol	8	BP
OuYang, 2002 [25]	102	Hypertension diagnostic criteria (unclear); TCM diagnostic criteria (unclear)	QJDH pill plus verapamil	Verapamil	8	BP; TCM-SSD; BL
Zhang, 1999 [26]	70	1999 WHO-ISH GMH; GCRNDTCM	QJDH pill	Nifedipine	8	BP
Ling, 2011 [27]	60	Hypertension diagnostic criteria (unclear)	QJDH pill	Captopril	4	BP
Liu, 2008 [28]	180	CGPMHBP-2000	QJDH pill plus Western drugs	Western drugs	24	BP
Zhong, 2006 [29]	80	Hypertension diagnostic criteria (unclear)	QJDH pill plus antihypertensive tablets	Antihypertensive tablets	4	BP
Zhou, 2002 [30]	80	Hypertension diagnostic criteria (unclear)	QJDH pill	Nifedipine controlled release tablet	4	BP

Abbreviations: QJDHW: Qi Ju Di Huang Wan; WHO-ISH GMH: WHO-ISH guidelines for the management of hypertension; GCRNDTCM: Guidelines of Clinical Research of New Drugs of Traditional Chinese Medicine; CGPMHBP: China Guidelines on Prevention and Management of High Blood Pressure; JNC-7: Seventh Report of the Joint National Committee on the Prevention, Detection, Evaluation, and Treatment of High Blood Pressure; TCM: traditional Chinese medicine; TCM-SSD: TCM syndrome and symptom differentiation; BP: blood pressure; BL: blood lipid; PV: plasma viscosity; Ang II: angiotensin II; ET: endothelin; CGRP: calcitonin gene-related peptide.

**Table 2:** Composition of different QJDHW preparations.

Study ID	Preparation	Composition
Li et al., 2012 [21]	Decoction	Chrysanthemum flower 20 g, Chinese wolfberry fruit 15 g, prepared rehmannia root 20 g, *Cornus* 15 g, common yam rhizome 15 g, poria 15 g, water plantain rhizome 12 g, tree peony bark 10 g, danshen root 15 g, earth worm 20 g, red peony root 12 g, two-toothed achyranthes root 20 g, gambir plant 12 g, and common self-heal fruit spike 15 g. Severe dizziness and tinnitus plus dragon bones 20 g and oyster shell 20 g; vexing heat in the five centers and red tongue plus common anemarrhena rhizome 12 g and Chinese wolfberry root bark 12 g; amnesia and lumbago plus Tortoise plastron 15 g, *Eucommia* bark 12 g, Chinese taxillus herb 15 g, and deer antler glue 10 g (melted in decoction).
Zhu, 2012 [22]	pill	Chinese patent medicine
Du et al., 2009 [23]	pill	Chinese patent medicine
Yang and Cheng, 2005 [24]	Modified QJDHW	Chrysanthemum flower 20 g, Chinese wolfberry fruit 15 g, prepared rehmannia root 20 g, *Cornus* 15 g, common yam rhizome 15 g, poria 15 g, water plantain rhizome 12 g, and tree peony bark 10 g.
OuYang, 2002 [25]	pill	Chinese patent medicine
Zhang, 1999 [26]	pill	Chinese patent medicine
Ling, 2011 [27]	pill	Chinese patent medicine
Liu, 2008 [28]	pill	Chinese patent medicine
Zhong, 2006 [29]	pill	Chinese patent medicine
Zhou, 2002 [30]	pill	Chinese patent medicine

Abbreviations: QJDHW: Qi Ju Di Huang Wan.

**Table 3:** Analyses of systolic blood pressure.

Trials		WMD [95% CI]	*P* value
*QJDHW versus * *antihypertensive drugs *			
QJDHW versus captopril	1	0.10 [−3.38, 3.58]	0.96

*Meta-Analysis *	1	0.10 [−3.38, 3.58]	0.96

*QJDHW plus antihypertensive drugs versus antihypertensive drugs *			
QJDH pill plus nifedipine controlled release tablet versus nifedipine controlled release tablet	1	−15.17 [−20.91, −9.43]	<0.00001
QJDH pill plus felodipine (plendil) versus felodipine (plendil)	1	0.32 [−4.16, 4.80]	0.89
Modified QJDHW plus metoprolol versus metoprolol	1	−5.00 [−20.44, 10.44]	0.53

*Meta-Analysis *	3	−5.52 [−8.96, −2.08]	0.002

Abbreviations: QJDHW: Qi Ju Di Huang Wan.

**Table 4:** Analyses of diastolic blood pressure.

Trials		WMD [95% CI]	*P* value
*QJDHW versus * *antihypertensive drugs *			
QJDHW versus captopril	1	−0.20 [−2.42, 2.02]	0.86

*Meta-Analysis *	1	−0.20 [−2.42, 2.02]	0.86

*QJDHW plus antihypertensive drugs versus antihypertensive drugs *			
QJDH pill plus nifedipine controlled release tablet versus nifedipine controlled release tablet	1	−14.06 [−17.85, −10.27]	<0.00001
QJDH pill plus felodipine (plendil) versus felodipine (plendil)	1	−7.00[−9.68, −4.32]	<0.00001
Modified QJDHW plus metoprolol versus metoprolol	1	−1.00 [−3.23, 1.23]	0.38

*Meta-Analysis *	3	−5.26 [−6.83, −3.70]	<0.00001

Abbreviations: QJDHW: Qi Ju Di Huang Wan.

**Table 5:** Analyses of TCM-SSD Scores.

Trials		Intervention (*n/N*)	Control (*n/N*)	RR [95% CI]	*P* value
*QJDHW versus * *antihypertensive drugs *					
QJDHW versus captopril	1	56/60	47/60	1.19 [1.03, 1.38]	0.02

*Meta-Analysis *	1	56/60	47/60	1.19 [1.03, 1.38]	0.02

*QJDHW plus antihypertensive drugs versus antihypertensive drugs *					
QJDH pill plus felodipine (plendil) versus felodipine (plendil)	1	31/36	24/36	1.29 [0.99, 1.8]	0.06
QJDH pill plus verapamil versus verapamil	1	31/34	18/34	1.72 [1.23, 2.40]	0.001

*Meta-Analysis *	2	62/70	42/70	1.48 [1.20, 1.82]	0.0003

Abbreviations: QJDHW: Qi Ju Di Huang Wan.
